# D-Limonene Is a Potential Monoterpene to Inhibit PI3K/Akt/IKK-α/NF-κB p65 Signaling Pathway in Coronavirus Disease 2019 Pulmonary Fibrosis

**DOI:** 10.3389/fmed.2021.591830

**Published:** 2021-03-09

**Authors:** Fan Yang, Ru Chen, Wan-yang Li, Hao-yue Zhu, Xiao-xuan Chen, Zhen-feng Hou, Ren-shuang Cao, GuoDong Zang, Yu-xuan Li, Wei Zhang

**Affiliations:** ^1^College of Traditional Chinese Medicine, Shandong University of Traditional Chinese Medicine, Jinan, China; ^2^Biomedical Research Institute of Fudan University, Shanghai, China; ^3^School of Public Health, Xiangya Medical College, Central South University, Changsha, China; ^4^College of Life Sciences, Shandong Normal University, Jinan, China; ^5^Department of Pulmonary Diseases, Affiliated Hospital of Shandong University of Traditional Chinese Medicine, Jinan, China; ^6^Second School of Clinical Medicine, Beijing University of Chinese Medicine, Beijing, China

**Keywords:** D-limonene, coronavirus disease 2019, coronavirus disease related pulmonary fibrosis, severe acute respiratory syndrome, PI3K/Akt signaling pathway

## Abstract

At the time of the prevalence of coronavirus disease 2019 (COVID-19), pulmonary fibrosis (PF) related to COVID-19 has become the main sequela. However, the mechanism of PF related to COVID (COVID-PF) is unknown. This study aimed to explore the key targets in the development of COVID-PF and the mechanism of d-limonene in the COVID-PF treatment. The differentially expressed genes of COVID-PF were downloaded from the GeneCards database, and their pathways were analyzed. d-Limonene was molecularly docked with related proteins to screen its pharmacological targets, and a rat lung fibrosis model was established to verify d-limonene's effect on COVID-PF-related targets. The results showed that the imbalance between collagen breakdown and metabolism, inflammatory response, and angiogenesis are the core processes of COVID-PF; and PI3K/AKT signaling pathways are the key targets of the treatment of COVID-PF. The ability of d-limonene to protect against PF induced by bleomycin in rats was reported. The mechanism is related to the binding of PI3K and NF-κB p65, and the inhibition of PI3K/Akt/IKK-α/NF-κB p65 signaling pathway expression and phosphorylation. These results confirmed the relationship between the PI3K–Akt signaling pathway and COVID-PF, showing that d-limonene has a potential therapeutic value for COVID-PF.

## Introduction

Since 2003, coronavirus has caused multiple major public health events that resulted in global epidemics, such as severe acute respiratory syndrome (SARS), Middle East respiratory syndrome (MERS), and coronavirus disease 2019 (COVID-19). Since December 2019 to date, SARS coronavirus 2 (SARS-CoV-2) has caused the most severe pandemic of *Coronaviridae* to date, but there is currently no specific drugs for COVID-19. Pneumonia is the main manifestation of COVID-19. In general, persistent inflammatory damage to lung tissue caused by various reasons develops into pulmonary fibrosis (PF) (1) and further leads to pulmonary dysfunction and reduced quality of life after recovery. Although PF changes are occasionally observed as sequelae of other respiratory viral infections, they appear to be more common after COVID (2). For example, in 1-year follow-up studies, PF was observed in the lungs of 27.5% of SARS survivors (*n* = 97) (3). Long-term follow-up studies have shown that many survivors of SARS-CoV infection show signs of fibrosis in their lungs (4–6). MERS coronavirus (MERS-CoV) infection can also be responsible for PF (7, 8). Pathological analysis revealed that the alveolar septum of MERS patients was destroyed and expanded, and type II alveolar epithelial cells proliferated and shed. The clinical manifestations and severity of COVID-19 are similar to those of SARS (9). A large amount of evidence supports that COVID-19 can contribute to PF (10). The pathological changes in the early lungs of COVID-19 can be manifested as viral interstitial pneumonia, suggesting that it is imperative to start anti-fibrosis treatment in the early clinical stage (11). Pirfenidone (PFD) is a commonly used drug for the clinical treatment of PF, but it cannot effectively prolong the survival of patients (12, 13). PFD has side effects of gastrointestinal reactions, rash, and photosensitivity (14, 15). At present, the mechanism of occurrence and development of COVID-related PF (COVID-PF) is not yet clear. Due to this lack of therapeutic options, there is a critical need to understand the molecular pathways involved in the development of COVID-PF (16, 17), thus helping to identify novel targets for therapy and develop new drugs.

Based on the research of GeneChip bioinformatics, sequence comparison, and cluster analysis are utilized to extract the biological information generated by gene chip technology. These endeavors will enable more comprehensive and systematic study for diseases. With a spurt of progress in high-throughput GeneChips and sequencing technologies in recent years, it has been made possible to reveal the gene expression profile of COVID-PF and the changes in PF tissue and cell key genes. On this basis, there have been successful examples in other fields to screen potential drugs by docking small molecular compounds with proteins according to their core differentially expressed gene proteins. d-Limonene is a terpenoid compound extracted from the essential oils of several citrus plants, and it is widely used in the food industry (17). Systematic reviews and pharmacological studies have found that d-limonene can prevent and control respiratory system damage through its anti-inflammatory and antioxidant activities (18). It has also been reported that d-limonene can improve the pulmonary tissue remodeling that occurs in animal models of pulmonary hypertension and asthma. Respiratory system damage and lung tissue structure remodeling are mutually causal and vicious circles and play a key role in forming COVID-PF. However, the mechanism of action of d-limonene on PF is unknown. This study aimed to analyze the key signaling pathways of the COVID-PF differentially expressed genes and explore the mechanism of d-limonene in a rat model.

## Materials and Methods

### Difference Analysis of Key Biological Processes and Signal Pathways Between Coronavirus Disease-Related Pulmonary Fibrosis and Pulmonary Fibrosis

GeneCards (https://www.genecards.org/) is a comprehensive database that integrates human genomic, transcriptomic, proteomic, clinical, and functional information. We used “corona virus disease related pulmonary fibrosis” and “pulmonary fibrosis” as keywords to search the key targets of COVID-PF and PF in the GeneCards database and import them into the Reactome Pathway Database (https://reactome.org/). The latter is a tool for comprehensive analysis and visualization of biological processes and signal pathways, which can compare the differences between COVID-PF and PF.

### Coronavirus Disease-Related Pulmonary Fibrosis Protein–Protein Interaction Construction, Gene Ontology, Pathway Enrichment, and Module Analysis

COVID-PF key target genes were imported into the STRING (https://string-db.org/) and Metascape (http://metascape.org/gp/index.html#/main/step1) platforms. Protein types were defined as *Homo sapiens* and screened to obtain protein interaction network diagrams and module analysis. The R software package was used to draw a bar graph to show the frequency of key target interactions in the network. Target genes were analyzed for Gene Ontology (GO) function and Kyoto Encyclopedia of Genes and Genomes (KEGG) pathway enrichment. GO functional analysis items with similar functions were clustered and constructed an interactive network, and the target pathway network was drawn based on the results of KEGG enrichment analysis.

### Molecular Docking

Based on the differential expression results of the above genes and proteins, and based on the pathway enrichment analysis, the core protein was selected for forwarding molecular docking with d-limonene. d-Limonene molecular structure data were obtained from the PubChem website (https://pubchem.ncbi.nlm.nih.gov/), and related protein crystal structure data from the RCSB website (http://www.rcsb.org/). We used Discovery Studio 2016 to calculate the molecular docking, and we drew 2D and 3D effect pictures. The results of molecular docking suggest the mechanism of the effect of d-limonene on COVID-PF and guided the selection of related detection indicators in subsequent animal experiments.

### Antibodies and Reagents

d-Limonene was purchased from Sigma-Aldrich Co., Ltd. (183164). Bleomycin (BLM) was obtained from Cool Chemical Technology (Beijing) Co., Ltd. (S656455V), and PFD was obtained from Beijing Continent Pharmaceutical Co., Ltd. (190806). The Masson Tricolor staining kit (G1006-100), H&E staining kit (GP1031), reactive oxygen species (ROS) test kit (ROS, 2019-07), and the detection kits for hydroxyproline (HYP; 201900711), malondialdehyde (MDA; 20190830), superoxide dismutase (SOD; 20191125), and total protein quantification [by bicinchoninic acid (BCA) method, 20190711] were all purchased from Nanjing Jiancheng Bioengineering Research Institute. Rabbit anti-phosphatidylinositol 3-kinase (PI3K) antibody (bs-10657R), rabbit anti-p-PI3K p110-Ser1070 antibody (bs-6417R), rabbit anti-AKT1 antibody (bs-0115R), rabbit anti-p-AKT1-S473 antibody (bs-12456R), and rabbit anti-p-NF-κB p65-S536 antibody (bs-0982R) were purchased from Biosynthesis Biotechnology Inc. (Beijing, China). Anti-IκBα kinase (IKK)-α antibody (A2062), rabbit anti-IκBα antibody (A1187), and anti-β-actin antibody (AC026) were obtained from Wuhan ABclonal Biotechnology Co., Ltd. Rabbit anti-p-AKT1-T308 antibody (GB13459), rabbit anti-nuclear factor-κB (NF-κB) p65 antibody (GB11997), rabbit anti-α-SMA antibody (GB11044), rabbit anti-COL-I-A1 antibody (COL1A1, GB11022-3), rabbit anti-COL-III antibody (COL3A1, GB13023-2), and horseradish peroxidase (HRP)-labeled goat anti-rabbit IgG antibodies (GB23303) were purchased from Wuhan Servicebio Technology Co., Ltd. The rat TGF-β1 ELISA kit was purchased from CUSABIO BIOTECH CO., Ltd. Rat interleukin (IL)-6, IL-1β, TNF-α, and VEGF ELISA kits were purchased from Wuhan Huamei Bioengineering Co., Ltd.

### Animal Grouping and Modeling

The study protocol was approved by the Research Ethics Committee of the Affiliated Hospital of Shandong University of Traditional Chinese Medicine (Approval No. AWE-2019-046) and met the National Institutes of Health Guide for the Care and Use of Laboratory Animals (NIH Publications No. 8023, revised 1978). Male Sprague Dawley (SD) rats [180–220 g, grade specific pathogen free (SPF)] were purchased from Jinan Pengyue Experimental Animal Breeding Co., Ltd. (Certificate No. SCXK[Lu]2014-0007, Jinan, China) and were placed in an environment with 12-h lighting and 12-h darkness per day and free feeding and drinking. After 7 days of adaptive breeding, the rats were randomly divided into six groups (six in each group): (1) saline (NS) group, (2) BLM + NS group, (3) BLM + d-limonene (25 mg/kg/day) group, (4) BLM + d-limonene (50 mg/kg/day) group, (5) BLM + d-limonene (100 mg/kg/day) group, and (6) BLM + PFD (150 mg/kg/day) group. The single intratracheal instillation of BLM (5 mg/kg) was used to induce PF in rats. After the modeling, the rats in the d-limonene group were injected intraperitoneally with the corresponding concentration of drugs. The rats in the PFD group were given intragastric administration of PFD and were sacrificed 28 days later. The rat blood, which was taken from the abdominal aorta, was separated at 5,000 rpm for 10 min at 4°C, and the serum was stored at −80°C. Lung tissues were collected and weighed. The formula lung index = (lung weight (g)/[body weight (g)] × 100% was calculated and obtained. The whole lung was lavaged three times using 2 ml of physiological saline, and bronchoalveolar lavage fluid (BALF) was then collected. Part of the lung tissues was placed in 4% paraformaldehyde, with the rest frozen in liquid nitrogen and stored at −80°C for further examination.

### Morphological and Histological Analyses

The lung tissues were fixed using 4% paraformaldehyde for 48 h, embedded in paraffin, and sliced with a thickness of 5 μm. The slices were stained with H&E and Masson trichrome to evaluate the pathological changes of lung tissues. At the same time, we obtained images that were magnified 200 times using an optical microscope. According to the Szapiel scoring standard and Ashcroft scoring standard, the degree of alveolitis and PF was scored, respectively (19, 20).

### Measurement of Hydroxyproline, Malondialdehyde, Reactive Oxygen Species Content, and Superoxide Dismutase Activity

The lung tissues were ground in cold physiological saline to obtain a 10% lung tissue homogenate. The homogenate was separated at 3,500 rpm 10 min at 4°C, and the supernatant was retained for the detection of HYP, MDA content, ROS level, and SOD activity. Both were determined according to the corresponding kit instructions.

### Enzyme-Linked Immunosorbent Assay

BALF and serum were prepared for ELISA. An ELISA kit was used to detect the contents of TNF-α, IL-1β, and IL-6 in rat BALF, and TGF-β1, and VEGF in serum. According to the manufacturer's instructions, samples were added to wells of an assay plate coated with a capture antibody. After incubation, the assay plate was washed three times, and a detection antibody was added. After 1-h incubation at room temperature and washing the plate three times, streptavidin–HRP was added to the wells. The color was developed with the tetramethylbenzidine substrate, and the absorbance was measured by enzyme-labeled instrument.

### Western Blot

After the total protein was extracted from the lung tissues using radioimmunoprecipitation assay (RIPA) lysis buffer containing 0.1% phenylmethylsulfonyl fluoride (PMSF), we extracted the protein from the lung tissues according to the manufacturer's instructions. We used the BCA protein detection kit to measure protein concentration. Equal amounts of protein samples were separated by sodium dodecyl sulfate–polyacrylamide gel electrophoresis (SDS-PAGE) and transferred to polyvinylidene fluoride (PVDF) membranes. After blocking, 5% non-fat milk in TBST was incubated with primary antibodies against PI3K (diluted 1:1,000), p-PI3K (diluted 1:800), AKT (diluted 1:1,000), p-AKT1-S473 (diluted 1:1,000), p-AKT1-T308 (diluted 1:1,000), NF-κB p65 (diluted 1:800), p-NF-κB p65 (diluted 1:1,000), IκBα (diluted 1:800), and IKK-α (diluted 1:800) at 4°C overnight. After being washed four times with TBST, we incubated with goat anti-rabbit secondary antibody for 1.5 h at room temperature and then washed four times with TBST, forming protein bands on the membrane with enhanced chemiluminescence reagent. ImageJ software was used to detect the gray value of the protein bands.

### Quantitative Real-Time Polymerase Chain Reaction

mRNA was extracted from the superficial dorsal horn using a universal RT-PCR Kit (Solarbio Science & Technology Co., Ltd., Shanghai, China) following the manufacturer's instructions. Samples were treated with DNase and then purified using an RNeasy kit (Qiagen, Hilden, Germany). Glyceraldehyde-3-phosphate dehydrogenase (GAPDH) was used as internal reference. PCR primer sequences included the following: IL-6: forward primer: 5′-ATGAAGTTTCTCTCCGCAAGAGACTTCCAGCCAG-3′; reverse primer: 5′-CTAGGTTTGCCGAGTAGACCTCATAGTGACC-3′, TNF-α: forward primer: 5′-CTCCCAGAAAAGCAAGCAAC-3′; reverse primer: 5′-CGAGCAGGAATGAGAAGAGG-3′, IL-1β: forward primer: 5′-ATGCCTCGTGCTGTCTGAC-3′; reverse primer: 5′-TCCCGACCATTGCTGTTTCC-3′, VEGF: forward primer: 5′-GGCTCTGAAACCATGAACTTTCT-3′; reverse primer: 5′-GCAGTAGCTGCGCTGGTAGAC-3′, NF-κB p65: forward primer: 5′-GACGAGGCTCGGAGAGCCCA-3′; reverse primer: 5′-CTGGGGCGGCTGACCGAATG-3′, PI3K: forward primer: 5′-TGCTATGCCTGCTCTGTAGTGGT-3′; reverse primer: 5′-GTGTGACATTGAGGGAGTCGTTG-3′, AKT: forward primer: 5′-GTGCTGGAGGACAATGACTACGG-3′; reverse primer: 5′-AGCAGCCCTGAAAGCAAGGA-3′, GAPDH: forward primer: 5′-TGATGACATCAAGAAGGTGGTGAAG-3′; reverse primer: 5′-TCCTTGGAGGCCATGTGGGCCAT-3′.

### Immunohistochemical Analysis

After being dewaxed, the lung slices were subjected to antigen recovery with citrate buffer under microwave heating. The slices were cooled down to room temperature and then sealed with 3% bovine serum albumin (BSA) for 30 min and were incubated overnight with primary antibody at 4°C. The primary antibodies used were rabbit anti-α-SMA antibody (diluted 1:1,000), rabbit anti-COL1A1 antibody (diluted 1:1,000), rabbit anti-COL3A1 antibody (diluted 1:200), rabbit anti-PI3K antibody (diluted 1:200), rabbit anti-AKT antibody (diluted 1:250), and rabbit anti-NF-κB p65 antibody (diluted 1:100). The slices were then washed with PBS and incubated with goat anti-rabbit secondary antibody (diluted 1:200) at 37°C for 50 min. After being rinsed with PBS, the slices were visualized with diaminobenzidine and counterstained with hematoxylin. The average optical density was measured using ImageJ.

### Statistical Analysis

Data are shown as mean ± standard deviation (SD). Differences between the groups were evaluated using one-way analysis of variance (ANOVA) followed by the least significant difference (LSD) *post-hoc* test. A *P* < 0.05 was considered statistically significant. Statistical analyses and figures were obtained using IBM SPSS Statistics 23.0 (IBM SPSS Software, NY, USA) and GraphPad Prism Version 8.0 (GraphPad Software, San Diego, CA, USA).

## Results

### The Difference Between the Key Biological Processes of Coronavirus Disease-Related Pulmonary Fibrosis and Pulmonary Fibrosis

**Figure 1:**
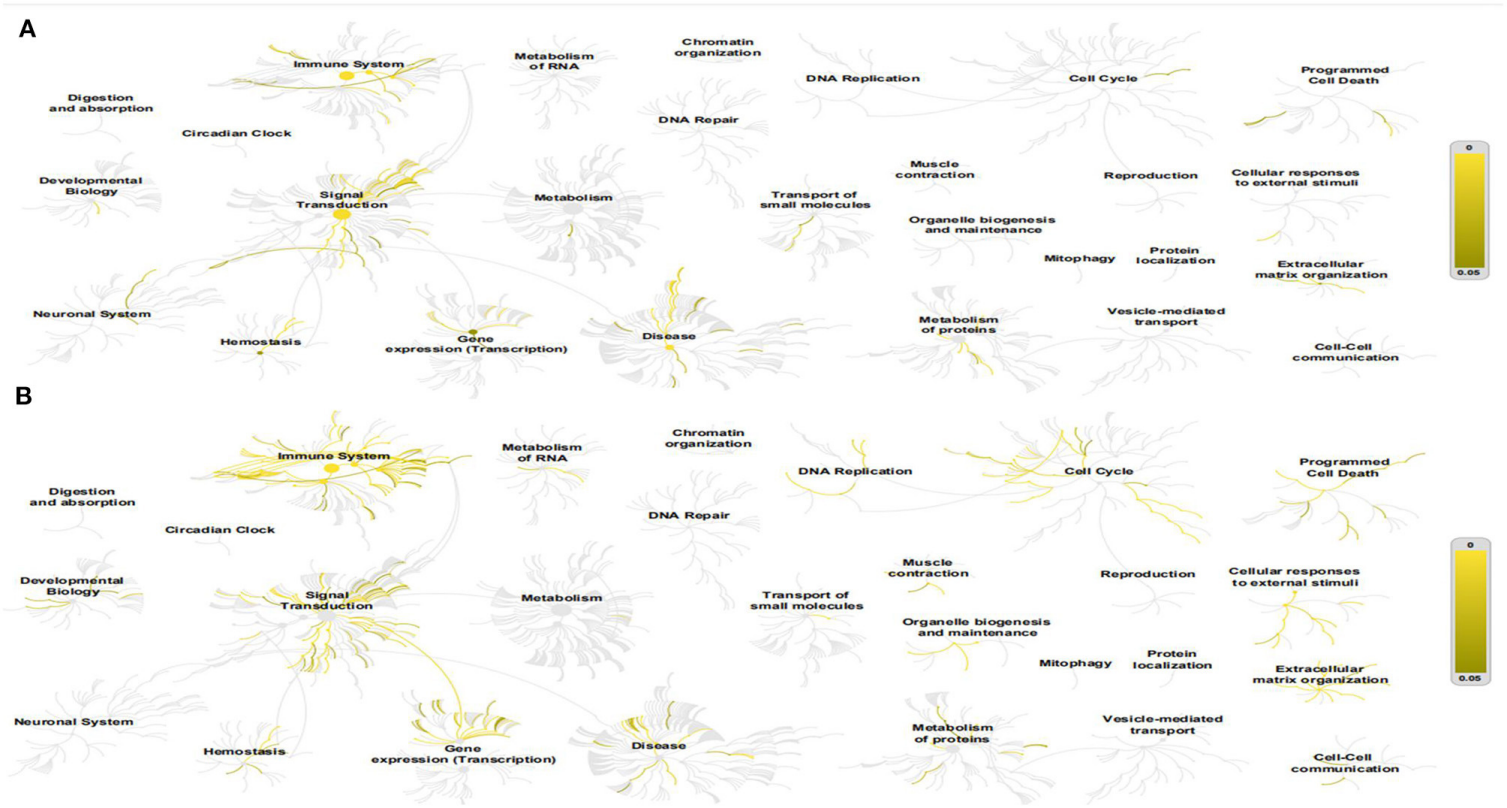
This figure shows a genome-wide overview of the results of coronavirus disease-related pulmonary fibrosis (COVID-PF) **(A)** and PF **(B)**. Reactome pathways are arranged in a hierarchy. The center of each of the circular “bursts” is the root of one top-level pathway. Each step away from the center represents the next level lower in the pathway hierarchy. The color code denotes the overrepresentation of that pathway in your input dataset. Light gray signifies pathways that are not significantly overrepresented.

The Reactome Pathway Database shows a genome-wide overview of the results of COVID-PF and PF (Figure 1). The first five core paths of COVID-PF are IL-10 signaling; constitutive signaling by aberrant PI3K in cancer; PI3K/AKT signaling in cancer; PI5P, PP2A, and IER3 regulate the PI3K/AKT signaling; and transcriptional regulation by the AP-2 (TFAP2) family of transcription factors. The first five core paths of PF are IL-4 and IL-13 signaling, signaling by ILs, cytokine signaling in immune system, immune system, and antigen processing–cross-presentation. This suggests that the biological pathways of COVID-PF and PF caused by other reasons are different. The PI3K/AKT signaling pathway plays a key role in the occurrence of COVID-PF.

### Protein–Protein Interaction Network Analysis, Gene Ontology Function Enrichment Analysis, and Kyoto Encyclopedia of Genes and Genomes Pathway Analysis

**Figure 2:**
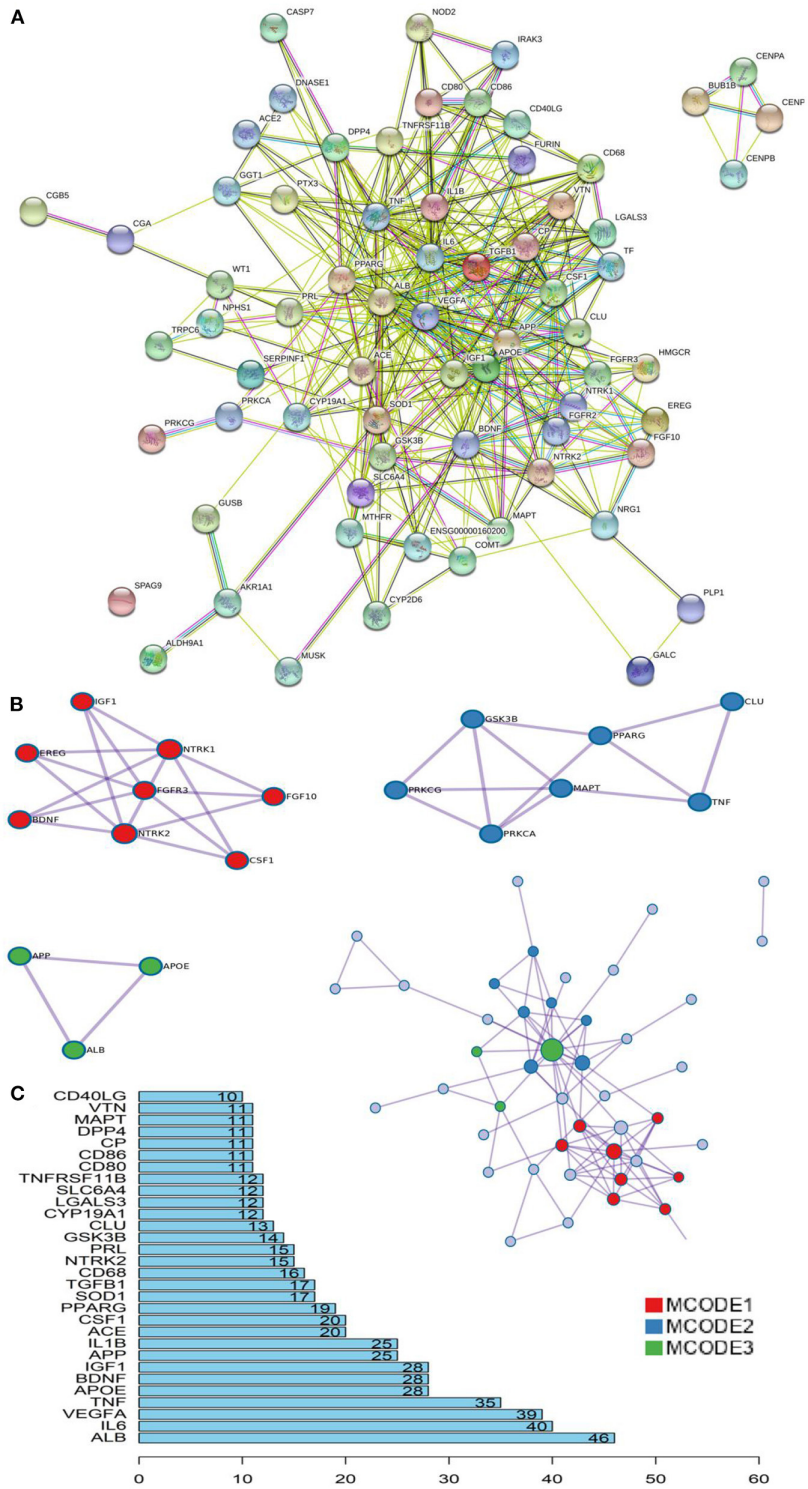
Key proteins and interactions in coronavirus disease-related pulmonary fibrosis (COVID-PF). **(A)** Protein–protein interaction (PPI) network of differential expression protein of COVID-PF. **(B)** Three important core modules and their relationship displayed in MCODE module analysis. **(C)** Ranking of correlation degree of key proteins.

**Figure 3:**
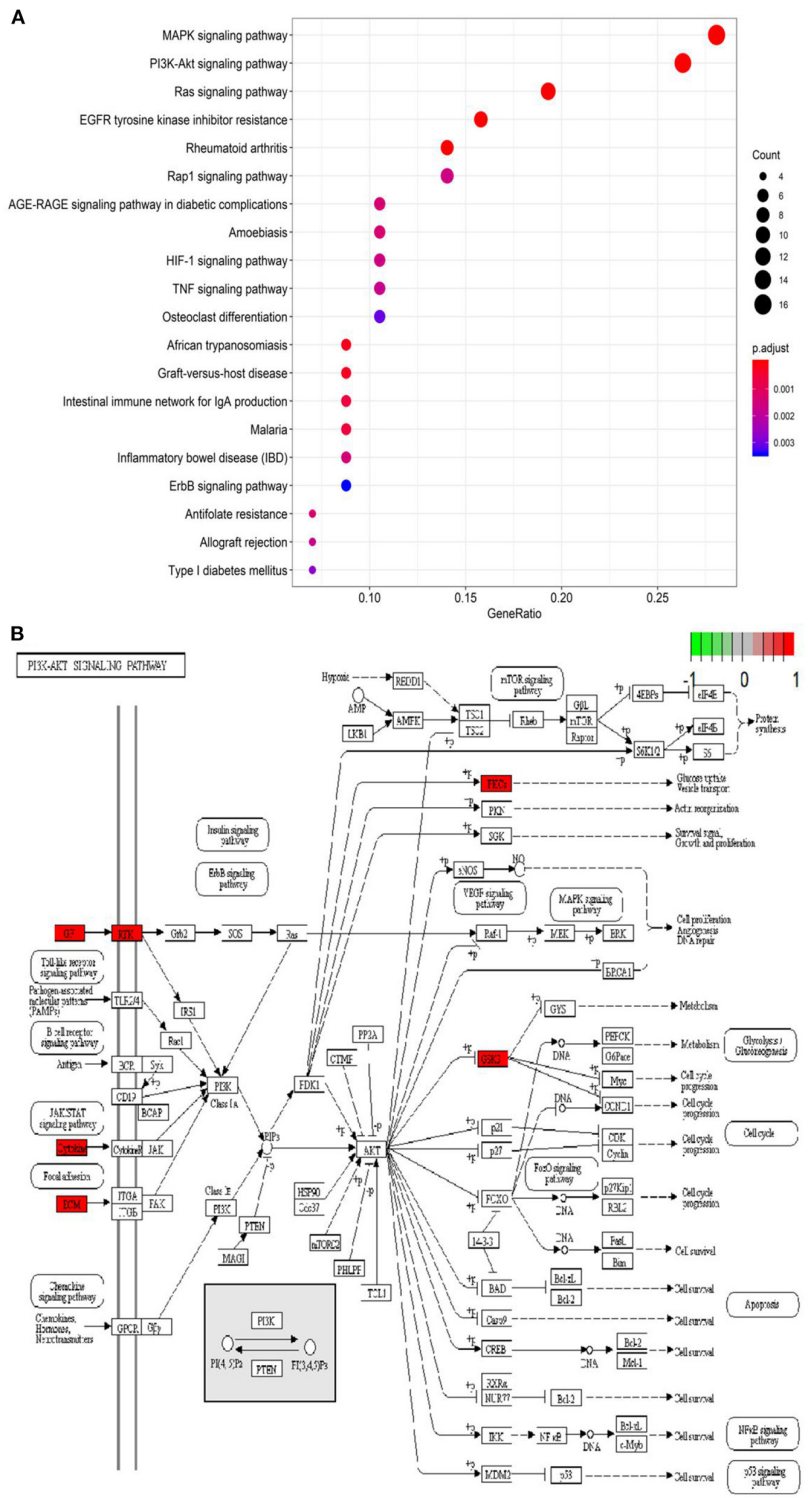
Kyoto Encyclopedia of Genes and Genomes (KEGG) pathway analysis. **(A)** Bubble chart of enrichment analysis results of KEGG pathway analysis. **(B)** The key target of the PI3K/AKT signaling pathway in coronavirus disease-related pulmonary fibrosis (COVID-PF).

Based on the STRING database and Metascape platform, a PPI network related to COVID-PF was constructed (Figure 2A). MCODE module analysis showed that the functions of three important core modules were mainly focused on vascular remodeling, inflammatory response, and PI3K/Akt signaling pathway-related proteins. The interaction between the three core modules is shown in Figure 2B. The sequence of the first 30 key proteins is shown in Figure 2C. The first five key proteins included ALB, IL-6, VEGFA, TNF, and APOE (Figure 2C). Biological process analysis suggested that the humoral immune response of COVID-PF patients was unbalanced, characterized by the high expression of inflammatory mediators (Supplementary Figure 1). KEGG pathway analysis revealed that the MAPK signaling pathway and PI3K/Akt signaling pathway were the core pathways of COVID-PF, which was consistent with the conclusion of the Reactome Pathway Database (Figures 3A,B).

### Molecular Docking

**Figure 4:**
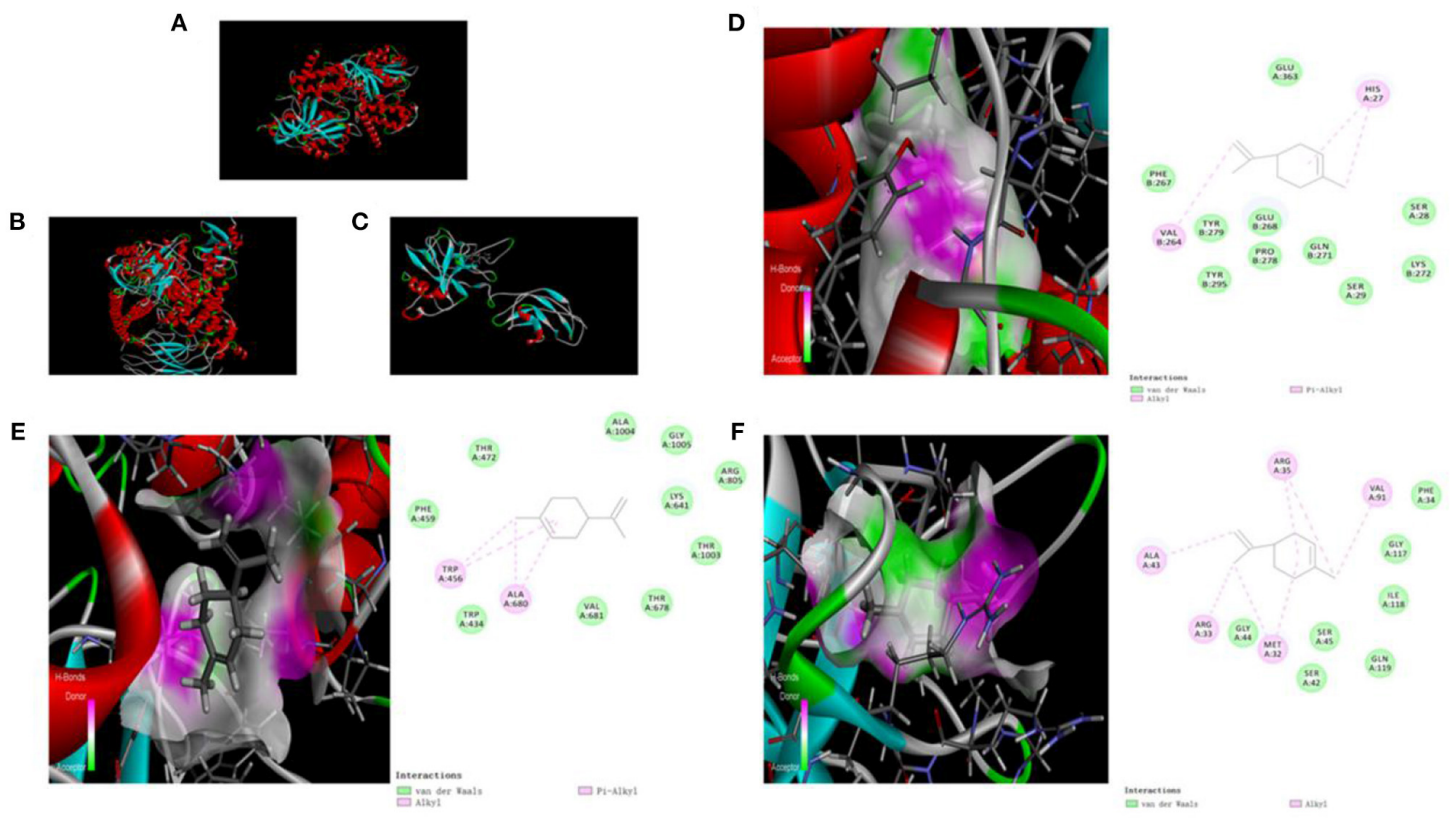
The upper left corner shows the 3D schematic diagram of d-limonene binding to PI3Kδ **(A)**, PI3Kβ **(B)**, and NF-κB p65 **(C)**. **(D)** 3D schematic diagram of active space binding of d-limonene and PI3Kδ. **(E)** 3D schematic diagram of active space binding of d-limonene and PI3Kβ. **(F)** 3D schematic diagram of active space binding of d-limonene and NF-κB p65.

We used molecular docking technology to dock key proteins suggested by PPI network analysis and KEGG pathway analysis with d-limonene. The CDOCKER experiment revealed that d-limonene can be docked effectively with PI3Kδ (PDB code: 4XEO), PI3Kβ (PDB code: 2Y3A), and NF-κB p65 (PDB code: 1VKX). Furthermore, the binding energies were −22.26, −14.54, and −18.77 kcal/mol. Molecular docking also revealed the binding sites of d-limonene and each protein, as shown in Figures 4A–F.

### D-Limonene Improves Bleomycin-Induced Pulmonary Fibrosis

**Figure 5:**
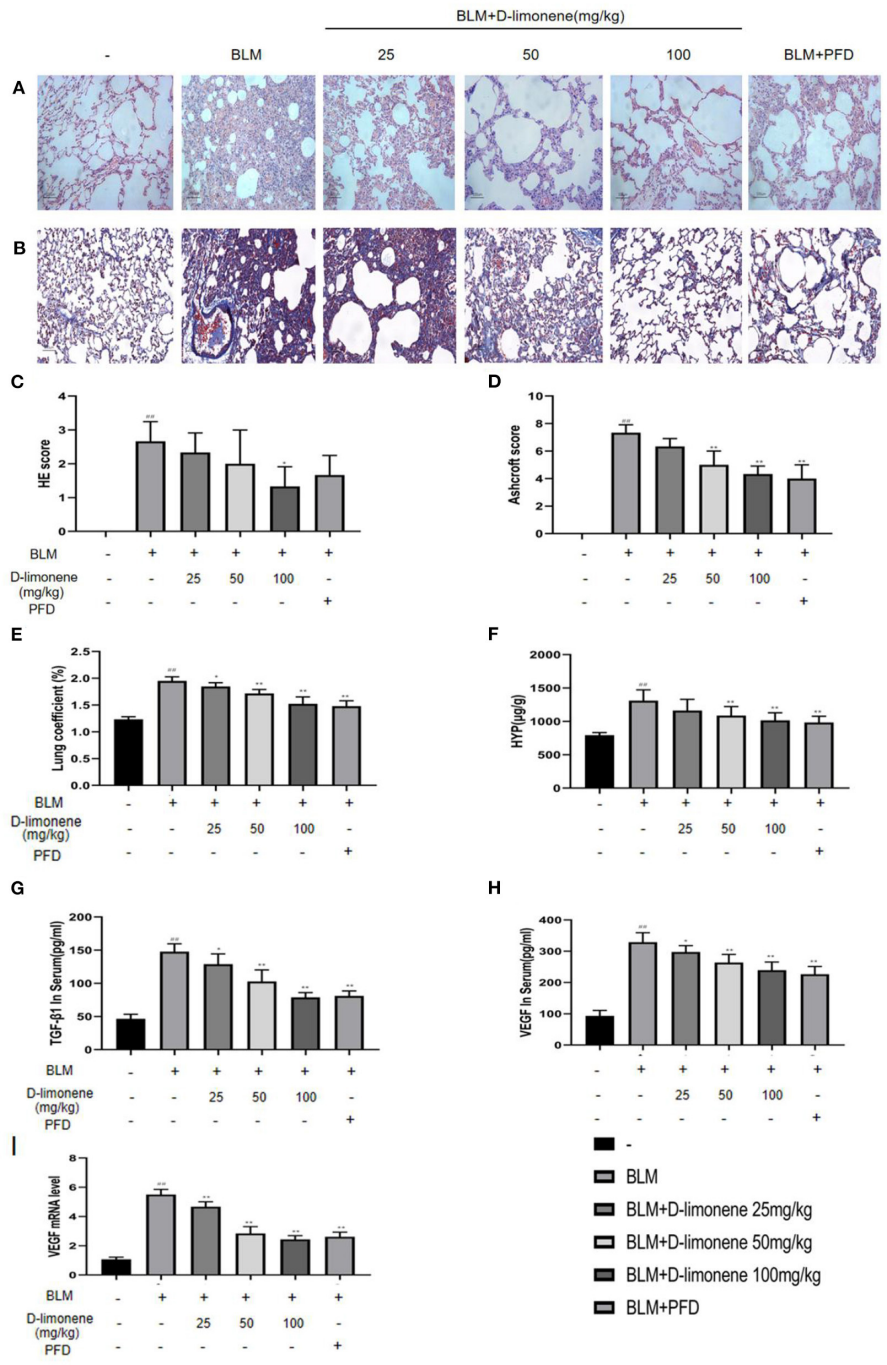
The protective effect of d-limonene on bleomycin (BLM)-induced pulmonary fibrosis (PF) in rats. **(A)** Photomicrographs of lung sections stained with H&E. **(B)** Photomicrographs of lung sections stained with Masson trichrome staining. **(C)** Alveolitis score of each group. **(D)** Statistics of the PF area in each group. **(E)** Measurement of lung coefficient in each group. **(F)** Determination of hydroxyproline content in lung tissues of each group. **(G)** Determination of TGF-β1 content in the serum of each group. **(H)** Determination of VEGF content in the serum of each group. **(I)** The mRNA expression levels of VEGF in each group detected by qRT-PCR. Data are presented as the means ± SD (*n* = 3 or 6), ^#^comparison with the control group, and *comparison with the BLM group. ^##^*P* < 0.01; *P < 0.05; and **P < 0.01.

PF was successfully induced by intratracheal instillation of BLM (5 mg/kg) in rats. H&E staining confirmed that the structure of rat lung tissues in the BLM group was disordered, with thickened alveolar walls; a large number of inflammatory cells had infiltrated the alveolar cavity and the interstitial fluid; and some of the alveoli disappeared. Masson trichrome staining revealed a large amount of collagen deposition (Figures 5A,B). However, d-limonene significantly improved lung tissue structural damage caused by BLM (Figures 5C,D). The protective effect of the 100 mg/kg dose group was similar to that of PFD (150 mg/kg), and the lung coefficient was significantly reduced in a dose-dependent manner (Figure 5E). HYP is the main component of collagen, VEGF is upregulated in lung fibroblasts under hypoxia, and TGF-β1 can induce the fibroblasts to synthesize a large amount of collagen. After treatment with d-limonene at a dose of 25–100 mg/kg, the levels of HYP in the lung tissues of lung fibrosis rats and the TGF-β1 in the serum were reduced compared with those in the BLM group. The expressions of VEGF and VEGF mRNA were downregulated (Figures 5F–I). There was no significant difference between the 100 mg/kg group and PFD (150 mg/kg) groups.

### D-Limonene Alleviates Pulmonary Fibrosis by Inhibiting the PI3K/Akt/IKK-α/NF-κB p65 Signaling Pathway

**Figure 6:**
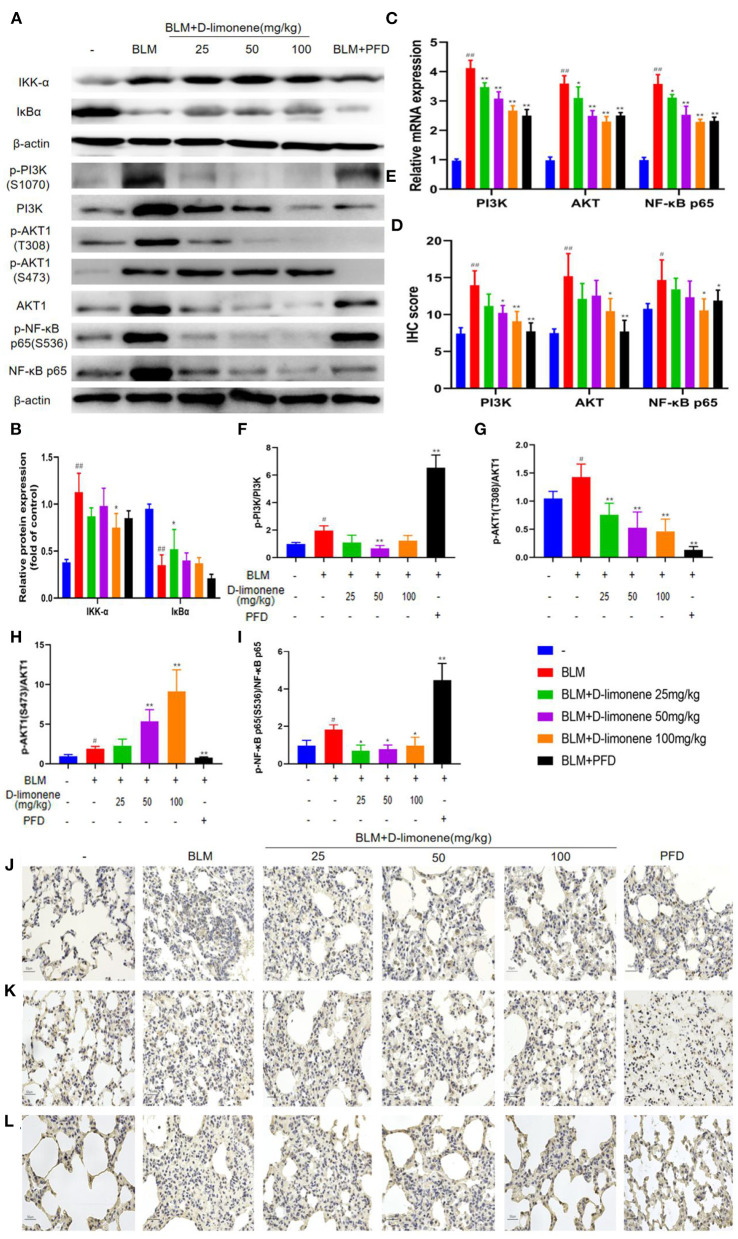
d-Limonene alleviates pulmonary fibrosis (PF) by inhibiting PI3K/Akt/IKK-α/NF-κB p65 signaling pathways. **(A)** Western blot analysis of the protein levels of IKK-α and IκBα in lung tissues. **(B)** Densitometric analysis of IKK-α and IκBα in the immunoblots, using β-actin as the internal reference. **(C)** The mRNA expression levels of PI3K, Akt, and NF-κB p65 in each group detected by qRT-PCR. **(D)** The average optical density of PI3K, Akt, and NF-κB p65. **(E–I)** Western blot analysis of PI3K **(F)**, Akt **(G,H)**, NF-κB p65 **(I)** protein expression, and phosphorylation level in lung tissue. **(J–L)** Immunohistochemical staining of PI3K- **(J)**, AKT- **(K)**, and NF-κB p65 **(L)**-positive cells in the lungs. Data are presented as the means ± SD (*n* = 3), ^#^comparison with the control group, and *comparison with the bleomycin (BLM) group. ^#^*P* < 0.05, ^##^*P* < 0.01; *P < 0.05; and **P < 0.01.

After BLM (5 mg/kg) induced the PF rat model successfully, the expressions of PI3K, Akt, IKK-α, and NF-κB p65 in the BLM group were all upregulated (*P* < 0.05), and IκBα expression was downregulated (*P* < 0.01), indicating that the PI3K/Akt/IKK-α/NF-κB p65 signaling pathway was activated (Figures 6A,B,E). After being administered PFD and different doses of d-limonene, the expressions of PI3K, Akt, IKK-α, and NF-κB p65 decreased in a dose-dependent manner, and the expression of IκBα was upregulated. In addition, the expressions of PI3K mRNA, Akt mRNA, and NF-κB p65 mRNA were downregulated (Figure 6C), indicating that d-limonene can inhibit the activation of the PI3K/Akt/IKK-α/NF-κB p65 signaling pathway in PF rats. The immunohistochemistry results also proved this (Figures 6D,J–L), and the effect of d-limonene at a dose of 100 mg/kg was similar to that of PFD. The study of phosphorylated proteins found that BLM induced the phosphorylation of PI3K in the rat model and also induced the rapid and sustained phosphorylation of AKT at Thr308, Ser473, and NF-κB p65 at Ser536. Different doses of d-limonene downregulated the expressions of p-PI3K, p-AKT Thr308, and p-NF-κB p65 Ser536 (Figures 6E–I). PFD downregulated the expressions of p-AKT Thr308 and p-AKT Ser473 (Figures 6G,H). However, we did not observe a significant change in p-AKT Ser473 expression in the d-limonene group (Figure 6H).

### D-Limonene Reduces Inflammatory Response, Oxidative Stress, and Collagen Deposition in Lung Tissue of Pulmonary Fibrosis Rats

**Figure 7:**
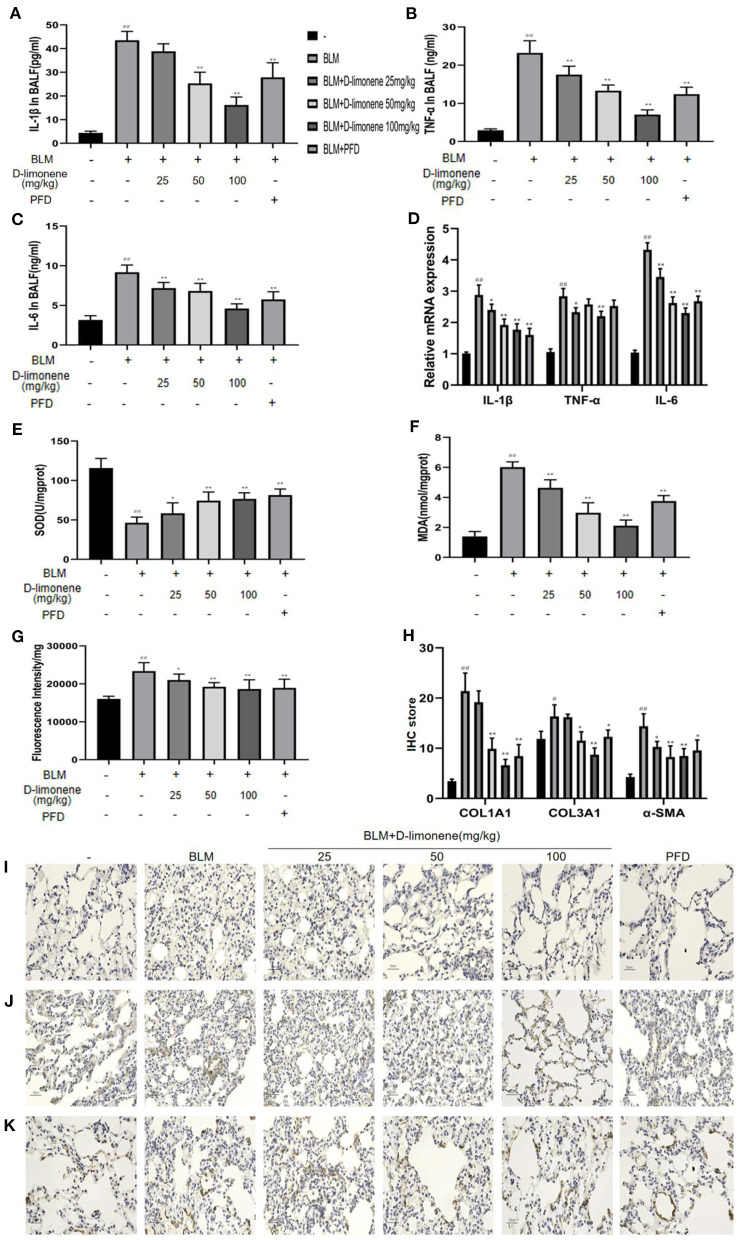
d-Limonene reduces inflammation, oxidative stress, and collagen deposition in the lung tissue of rats with pulmonary fibrosis (PF). **(A)** Determination of IL-1β in bronchoalveolar lavage fluid (BALF) of each group. **(B)** Determination of TNF-α in BALF of each group. **(C)** Determination of IL-6 in BALF of each group. **(D)** The mRNA expression levels of IL-1β, TNF-α, and IL-6 in each group detected by qRT-PCR. **(E)** Determination of superoxide dismutase (SOD) in the lung tissue of each group. **(F)** Determination of malondialdehyde (MDA) in the lung tissue of each group. **(G)** Determination of reactive oxygen species (ROS) in the lung tissue of each group. **(H)** The average optical density of COL1A1, COL3A1, and α-SMA. **(I,K)** Immunohistochemical staining of COL1A1 **(I)**, COL3A1 **(J)**, and α-SMA **(K)**-positive cells in the lungs. Data are presented as the means ± SD (*n* = 3 or 6), ^#^comparison with the control group, and *comparison with the bleomycin (BLM) group. ^#^*P* < 0.05, ^##^*P* < 0.01; *P < 0.05; and **P < 0.01.

In order to verify the anti-inflammatory and anti-oxidant capacity of d-limonene, we used ELISA to detect relevant biomarkers. As expected, 28 days after successful modeling, there were increases in the levels of inflammatory mediators and oxidative stress in the lung tissue of the BLM Group (*P* < 0.01). At the same time, d-limonene reduced the levels of IL-1β, TNF-α, IL-6, and MDA in the lung tissues of PF rats and ROS levels; downregulated the expressions of IL-1β mRNA, TNF-α mRNA, and IL-6 mRNA; and increased the activity of SOD. The effects of 100 mg/kg d-limonene and PFD groups were similar or significantly different (Figures 7A–G). Collagen deposition is a significant manifestation of PF. Immunohistochemistry showed that the expressions of COL1A1, COL3A1, and α-SMA were upregulated in the BLM group (*P* < 0.05), while PFD and d-limonene decreased their expressions. The effect of d-limonene was more obvious than that of PFD, and it was dose-dependent (Figures 7H–K).

## Discussion

In this study, the key biological processes and signaling pathways of COVID-PF differentially expressed genes were analyzed. The mechanism of action of d-limonene in a rat PF model was discussed. We found that the occurrence of COVID-PF is closely related to the PI3K/AKT signaling pathway. d-Limonene can significantly improve the collagen deposition and oxidative stress levels of PF rats and inhibit inflammation and angiogenesis. The mechanism may be mediated by inhibiting the PI3K/Akt/IKK-α/NF-κB p65 signaling pathway.

As a “global pandemic” disease announced by WHO, the number of COVID-19-related infections continues to rise. Coronavirus not only has a higher fibrogenic potential than common respiratory viruses but also makes patients more likely to enter a dangerous acute respiratory distress syndrome (ARDS) state. Patients with ARDS require mechanical ventilation to maintain respiratory function during treatment to improve the patient's hypoxic state, and mechanical ventilation-related lung injury is a major adverse reaction caused by the ventilator to patients (21). The harmful effect of mechanical ventilation is not only mediated by the systemic release of local inflammatory cytokines but also induced by mechanical stress, which can lead to the transformation of epithelial stroma and the release of fibrogenic mediators caused by cell stretching and mechanical ventilation, which then develops into PF (22, 23). Therefore, the factors of continuous lung damage in the course of COVID-19 are complicated, and PF may become the main complication after the completion of this outbreak. Bioinformatics research suggests that inflammatory response, oxidative stress, angiogenesis, and other biological processes and the PI3K/AKT signaling pathway are closely related to COVID-PF. The PI3K/AKT signaling pathway is involved in many cellular processes such as cell differentiation, proliferation, apoptosis, and angiogenesis (24–26). Studies have shown that the PI3K/Akt pathway, as a form of “adaptive strategy,” is involved in the immune response process of the host cell to counteract viral invasion (27). This partly explains the preference of COVID-PF as a complication and sequela of viral infection for PI3K/Akt signaling pathway.

Although COVID is not unique to humans, animal models of coronavirus infection show different disease characteristics than humans. Although several other animal models of SARS-CoV infection have been described, these models rarely show lethality (28–32). Although studies have predicted that the survival of lethally infected aged mice could be extended to the fibrotic phase of ARDS using sublethal infection, the model has clear fibrosis characteristics, which are very different from the clinical manifestations of human patients (33). Therefore, this study selected BLM to induce PF in rats at the start of the study. Our study found that the PI3K/Akt signaling pathway in the lung tissue of PF rats was significantly activated, consistent with the predictions of bioinformatics. AKT is the main effector kinase of the PI3K signaling pathway. AKT activated by phosphorylation has important biological significance. Many growth factors, hormones, and cytokines activate AKT by binding their homologous receptor tyrosine kinase or by triggering the activation of lipid kinase PI3K, thereby generating PIP3 in the plasma membrane of the cell. AKT binds to PIP3 through its PH domain, causing AKT to translocate to the cell membrane and phosphorylated by the double phosphorylation mechanism. PDK1, which is also translocated to the cell membrane due to its PH domain, can also phosphorylate AKT by activating the Thr308 site. The secondary phosphorylation of Ser473 at the carboxyl terminus of AKT is also necessary for activity and is performed by mTORC2 (34, 35). Studies have confirmed that PI3K/Akt signaling pathway is involved in the pathogenesis of PF, and blocking the PI3K/Akt pathway can reduce BLM-induced inflammation and fibrosis (36). NF-κB is a nuclear transcription factor that is involved in the expression of inflammatory cytokine genes. At rest, the NF-κB dimer binds to IκBα and exists in the cytoplasm in an inactive form. Under the stimulation of lipopolysaccharide (LPS) and TNF-α (37), IKK is activated to phosphorylate serine residues at specific sites of IκBα and then ubiquitinates and degrades. At the same time, Ser536 of NF-κB p65 transcription activation domain can also be phosphorylated by IKK to enhance its transcriptional activity. In contrast, the process of LPS activation of NF-κB p65 is initiated by upstream kinases such as PI3K/AKT (38, 39). This study found that d-limonene can dose-dependently inhibit the expression of PI3K, Akt, and NF-κB p65; can inhibit the upregulation of IKK-α and the degradation of IκBα; and can inhibit the phosphorylation of PI3K, AKT (Thr308 site), and NF-κB p65 (Ser536 site), which is consistent with previous reports on the mechanism of action of d-limonene (40–42).

It has been reported that a hypoxic environment can promote the progression of fibrosis through epithelial–stromal transformation. Furthermore (43), hypoxia not only can directly cause lung tissue damage but also can aggravate the inflammatory response and oxidative stress. In the inflammatory state, the production of oxygen free radicals increases, and the body cannot produce enough SOD and catalase to eliminate them in time, which aggravates the damage, as mentioned above (44). Moreover, activation of the PI3K/AKT cascade is triggered by ROS (45). Our study found that d-limonene can effectively inhibit the secretion of inflammatory mediators and reduce the level of oxidative stress, which is consistent with previous reports (46). Hypoxia is the inevitable state of ARDS. In the middle and late stages of ARDS, with the initiation of lung injury repair mechanism, collagen deposition, and fibrosis promotion level increase (47). TGF-β, a major fibrogenic factor, is also one of the promoters of the PI3K/Akt signaling pathway (48). In this process, the necessary vascular remodeling and generation processes are very significant in COVID-PF. Some studies have shown that epidermal growth factor receptor (EGFR) signaling is a key regulator of SARS-CoV-induced lung damage leading to fibrosis (49) and mainly regulated by the PI3K/Akt signaling pathway. d-Limonene can downregulate the expression of fibrotic markers such as COL1A1, COL3A1, and α-SMA and can reduce the content of TGF-β1 and VEGF mRNA in tissues, which is of positive significance for COVID-PF and even ARDS in the middle and late stages. Although there are inherent difficulties in preparing animal models of COVID-PF as mentioned above, this study did not proceed directly on the relevant animal models, so only d-limonene can be called a “potential” effective natural compound. In addition, there are few studies on COVID-PF at present. The differential gene expression data collected in the public database may not fully reflect all the characteristics of COVID-PF. Moreover, due to factors such as lack of time, we have not yet established a sufficient number of pathologically confirmed COVID-19-related PF patient databases to support our research conclusions. The above items are the main limitations of this research. In the current imaging analysis of these patients, we found that their PF can show two patterns of usual interstitial pneumonia or non-specific interstitial pneumonia—these are two completely different outcomes. Therefore, studying the pathological characteristics and prognosis of COVID-19-related PF in different populations and the therapeutic value of existing drugs for other fibrotic lung diseases on COVID-19-related PF should be the next research direction. Therefore, in future studies, we will continue to pay attention to the research progress of this disease in order to fully understand its pathogenesis.

## Conclusions

It is proved that the imbalance between collagen breakdown and metabolism, inflammatory response, and angiogenesis are the core processes of COVID-PF, and PI3K/AKT signaling pathways and related signal transduction molecules are the key targets of the COVID-PF treatment. The ability of d-limonene was reported for the first time to protect against lung fibrosis induced by BLM in rats. The mechanism is related to the binding of PI3K and NF-κB p65 and the inhibition of PI3K/Akt/IKK-α/NF-κB p65 signaling pathway expression and phosphorylation. Additionally, new insights are provided into the potential value of d-limonene in the treatment of COVID-PF. However, at this time, the research on the differential gene expression of COVID-19 leaves some room for improvement, so our research on COVID-PF cannot fully summarize the characteristics of COVID-19-PF. It remains to be further explored.

## Data Availability Statement

The datasets presented in this study can be found in online repositories. The names of the repository/repositories and accession number(s) can be found in the article/Supplementary Material.

## Ethics Statement

The animal study was reviewed and approved by Research Ethics Committee of the Affiliated Hospital of Shandong University of Traditional Chinese Medicine.

## Author Contributions

Experimental design was carried out by FY and WL. Validation was performed by ZH and RCa. Data curation was done by XC and HZ. Writing-original draft preparation was done by GZ and YL. Writing-review and editing was performed by RCh and WZ. All authors contributed to the article and approved the submitted version.

## Conflict of Interest

The authors declare that the research was conducted in the absence of any commercial or financial relationships that could be construed as a potential conflict of interest.
