# Supplementary figures and images for: D-Limonene Is a Potential Monoterpene to Inhibit PI3K/Akt/IKK-α/NF-κB p65 Signaling Pathway in Coronavirus Disease 2019 Pulmonary Fibrosis
Source: Front Med (Lausanne). 2021 Mar 9;8:591830. doi: 10.3389/fmed.2021.591830 (PMC7985179; doi:10.3389/fmed.2021.591830)

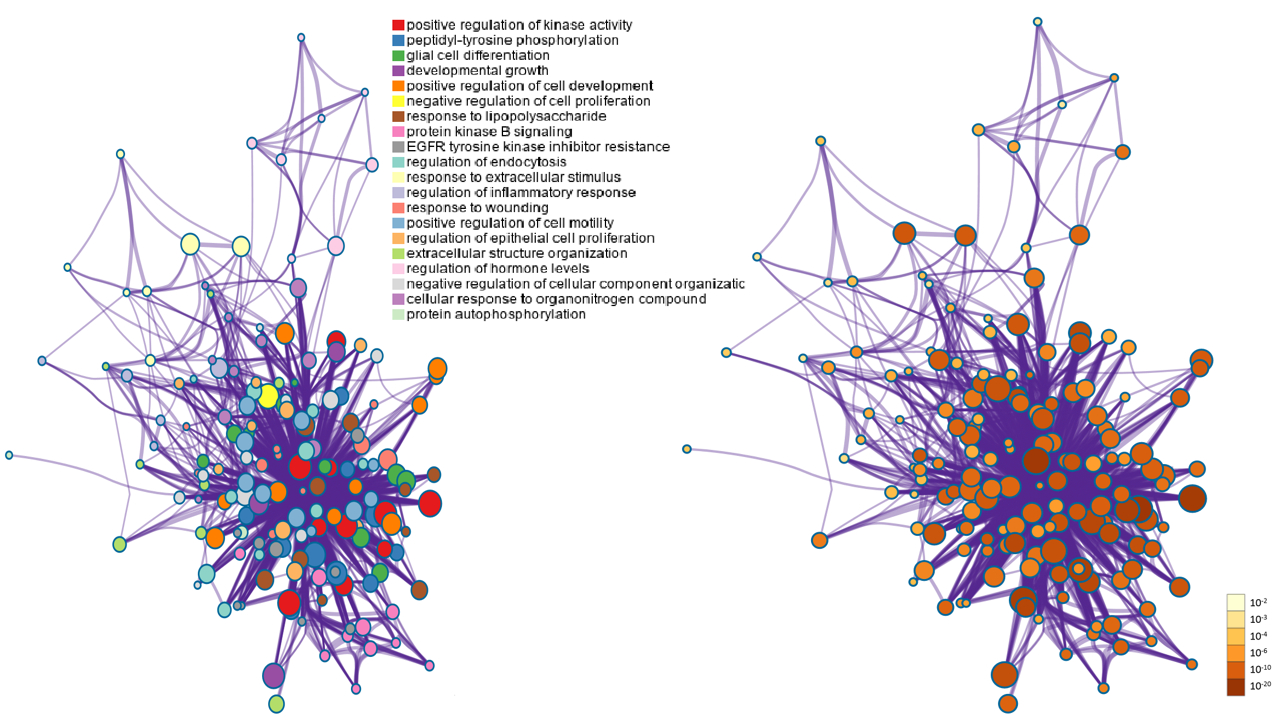

Supplement: Supplementary file 1 [file Image_1.TIF]
